# Professional Interpreter Services and the Impact on Hospital Care Outcomes: An Integrative Review of Literature

**DOI:** 10.3390/ijerph20065165

**Published:** 2023-03-15

**Authors:** Michelle Kwan, Zakia Jeemi, Richard Norman, Jaya A. R. Dantas

**Affiliations:** Curtin School of Population Health, Curtin University, Perth 6102, Australia

**Keywords:** language barriers, professional interpreter services, hospital settings, culturally and linguistically diverse patients

## Abstract

Migration patterns have rapidly changed in Australia and elsewhere, which have contributed to increasingly culturally and linguistically diverse societies. This requires healthcare sectors to provide professional interpreter services for patients with a language barrier to eliminate healthcare disparities. This integrative review aimed to investigate the impact of professional interpreter services on hospital care outcomes and the associated cost of service provision. A systematic search of five databases was conducted for peer-reviewed articles from January 1996 to December 2020. Data were extracted for the hospital setting, intervention, population, study design, outcomes and key findings. Following the PRISMA guidelines, full-text screening identified 37 articles that were analysed and included. Communication quality, hospital care outcomes and hospital costs were the three main themes identified. Closing the language gap should be a primary consideration to prevent adverse events that affect patient safety and the standard of care in hospitals. The findings of this review indicate the provision of professional interpreter services can enhance hospital care for linguistically diverse patients by improving patient–provider communication. To gain insight into the changing patterns on the outcomes of medical care, further research requires efforts by the hospital administrative system to document complete records of service usage.

## 1. Introduction

The negative effects of language barriers in the hospital setting have been widely documented in the global literature [1,2,3,4,5]. Existing findings have illustrated that patients receiving “language discordant care” are more prone to adverse events and potentially life-threatening conditions at different stages of hospital care including delay in treatment diagnosis at admission, poor communication for surgical procedure and at discharge which inevitably lead to hospital readmissions and an increase in healthcare costs [1,2,3]. This is concerning to nations with a growing culturally and linguistically diverse population. For instance, in Australia, the 2016 Census revealed that the proportion of the overseas-born population coming from non-English-speaking backgrounds has increased since 2011 and more than one-fifth of Australians (21%) spoke languages other than English at home including Mandarin, Arabic, Cantonese and Vietnamese [6]. This is an indication of the growing cultural and linguistic diversity in Australia and the importance of addressing the linguistic needs of individuals with limited English proficiency (LEP). In English-speaking countries such as Canada, the UK and the US, poor English proficiency hinders an individual’s ability to interact within the health system, limiting their access to health services, which in turn increases health disparities [7].

A worldwide strategy to bridge the language gap is the provision of professional interpreter services in hospitals. In Australia, Canada, the US and the UK, language service policies, standards and guidelines have been developed to mandate the use of interpreter services [7,8,9,10]. However, trends of underuse are evident across the literature revealing the relatively high use of ad hoc interpreters such as family, friends and untrained interpreters, and the challenges of engaging with a professional interpreter [11,12]. In some instances, bilingual providers adopt the interpreter role and may lack the skills of interpreting complex medical terminology [13]. Inappropriate language assistance also impacts interpretation quality which could lead to potential clinical consequences [14,15]. Thus, strengthening the provision of professional interpreter services in hospitals is crucial to facilitate communication between healthcare providers and patients with a language barrier.

The aim of this integrative review was to explore the global literature on the impact of professional interpreter services on hospital care to understand the effectiveness of the intervention within the hospital setting. The literature that provides cost evidence of interpreter services was also explored to identify potential cost benefits to the health system. As all health systems face budgetary constraints, further examination into the effectiveness of interpreter services in the hospital setting is needed to increase funding support and to inform policy changes. This integrative review is part of a larger study that explored interpreter service usage at a Western Australian (WA) hospital, located in Perth, providing hospital and community care to meet the broader health needs of the population. The results of the larger study including the integrative review are published under a Creative Commons license in a report by Kwan et al. 2020 [16].

## 2. Materials and Methods

The methodological approach reported in this integrative review followed a systematic format proposed by the Preferred Reporting Items for Systematic Reviews and Meta-Analyses (PRISMA) [17]. Adopting a systematic approach enabled a detailed search to identify and summarise the available evidence, and to examine the impact of using interpreter services on hospital care and patient outcomes, and the associated cost of service provision.

### 2.1. Search Strategy

Five electronic databases including EBSCO, MEDLINE, ProQuest, PubMed and Scopus were searched for peer-reviewed articles. A Boolean search was applied on the following combination: “Communication Barriers” OR “limited English proficiency” AND “interpret* services” AND “Quality of Health Care/” OR “length of stay and readmissions” OR “patient satisfaction” OR “hospital cost”. Search terms were meshed to subject headings based on specific database searching.

All searches were limited to the English language for this study. The references were managed and recorded using the reference management software EndNote X9. Only studies that met the inclusion and exclusion criteria were included in the review (Table 1).

### 2.2. Search Outcomes

**Figure 1:**
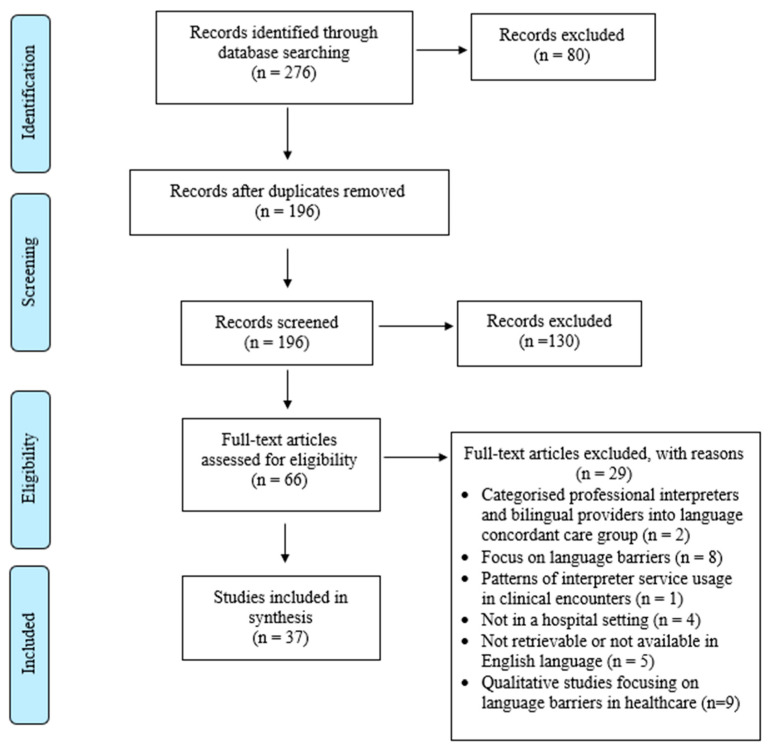
PRISMA flow chart presenting the screening and selection process of studies.

The initial database search of papers published between 1996 and 2020 yielded 276 articles (Figure 1). After the removal of duplicates, 196 articles remained for title and abstract screening. Two reviewers independently screened the titles and abstracts which excluded 130 articles for not meeting the inclusion criteria. Sixty-six papers remained for the full-text screening, and we excluded twenty-nine papers for not meeting the inclusion criteria. Qualitative studies were excluded from this review as they focus on patient encounters with language barriers in healthcare. In total, 37 papers were selected and included in the review for quality assessment and data analysis.

### 2.3. Data Synthesis and Analysis

The inclusion of diverse sources presented a challenge for quality appraisal in this review. With no gold standard of evaluating primary sources in integrative reviews, a quality assessment was not undertaken. Rather, data were abstracted based on the “authenticity, informational value, and representativeness” of primary sources [18]. The data synthesis followed the stages described by Whittemore and Knafl (2005), including data reduction, data display, data comparison and conclusion drawing from verification [18].

The process involved extracting data onto a table which included the following items: author/year/country, hospital setting, study design, types of interpreter service(s) and comparator, sample characteristics, outcome(s) and key findings. A narrative synthesis was conducted to arrive at conceptually coherent themes and subthemes. Study variables were organised into ten outcome categories and were then placed into conceptually coherent themes according to the review objectives. Three themes were derived which included the communication quality between patients and healthcare providers, hospital care outcomes and hospital cost (see Table 2).

## 3. Results

### 3.1. Characteristics of Studies

Most of the studies were quantitative studies (n = 36) and only one used mixed methods. Of the thirty-seven studies included in this review, thirty studies were conducted in the US, six in Australia and one in Sweden. The sample population included families or patients with LEP or with a lack of language proficiency in the host country, with a primary language not from the host country.

Studies were conducted in various hospital settings: outpatient clinics (n = 9); emergency department (ED) (n = 2) and paediatric ED (n = 7); inpatient ward (n = 3); both ED and inpatient ward (n = 1); primary care clinic and ED (n = 1); general or paediatric hospital settings (n = 3); rehabilitation hospital (n = 1); large metropolitan facility (n = 1); medicine or surgical floors (n = 4); an obstetric and gynaecological unit (n = 1); tertiary care (n = 2); internal medicine clinic (n = 1); and infection diseases service (n = 1). Organisation of the results was similar to the systematic review by Karliner et al. [19], where outcomes were grouped into major themes, and when multiple outcomes appeared, these were grouped according to their outcome category.

### 3.2. Theme 1: Communication Quality between Patients and Healthcare Providers

This theme illustrates the importance of using professional interpreter services to improve the communication quality between patients and healthcare providers, which includes accuracy of interpretation and language comprehension. More details are provided in Table 3.

#### 3.2.1. Interpretation Errors

The persistent use of ad hoc interpreters such as friends or family members can have significant negative consequences for patients with a language barrier. Five US studies provided supporting evidence suggesting that professional interpreter services resulted in fewer interpretation errors with potential clinical consequences compared to ad hoc interpreters and no interpreter use [14,15,20,21,22]. Using audiotaped transcribed clinical encounters, omission errors (uninterpreted words/phrases) were the most common interpretation errors, particularly when using ad hoc interpreters or in encounters without interpretation use [14,15]. One study reported that healthcare providers were more likely to commit false fluency (76%) in encounters with a hospital interpreter present: 58% of these occurred when an interpreter was absent from the room or telephone interpretation, and 42% of errors occurred when providers were not corrected by the interpreter [20].

Two studies compared remote simultaneous medical interpreting (RSMI—a form of remote interpretation provided within milliseconds of the original speech) to the traditional interpretation method (remote consecutive medical interpretation, in-person interpretation and ad hoc interpretation) and found that RSMI resulted in fewer interpretation errors [21,22]. This finding may be due to the simultaneous nature of the mode of interpretation where interpretation is provided immediately after speech and does not require interpreters to recall a large amount of information [21,22].

When comparing interpretation modalities, there was no consensus on which mode provided the highest quality of interpretation. Rather, professional interpreters who trained longer than 100 h committed a significantly lower proportion of errors with clinical consequences compared to interpreters who were trained for fewer than 100 h (2% vs. 12%, *p* = 0.03) [14]. Regardless of interpretation types, the overall findings would suggest that using professional interpreter services reduced interpretation errors with clinical consequences.

#### 3.2.2. Language Comprehension

Conducted within the paediatric hospital setting, three US studies assessed parents’ understanding of their child’s diagnosis using self-reported measures [23,24,25]. In family-centered rounds where parents were invited into the medical decision-making process, one study reported that videoconferencing and in-person medical interpreters assisted with parents’ understanding of their child’s medical condition [23]. Another study compared remote interpretation modalities (telephone and videoconferencing) and found that parents using videoconferencing were significantly more likely to recall a child’s diagnosis compared to those using telephone interpretation (*p* = 0.03) [25]. With contrasting findings, one study compared professional interpreter services to bilingual providers and found no differences between the interpretation types on family comprehension of the paediatric diagnosis [24].

### 3.3. Theme 2: Hospital Care Outcomes

Safe routine care in the hospital setting requires clear and effective communication between patients and healthcare providers. This theme focuses on outcomes related to the hospital care process when professional interpreter services are used. More details are provided in Table 4.

#### 3.3.1. Visit Length and Throughput Times

Efficient patient flow is crucial in the hospital setting to ensure all patients receive timely care. Examining the visit length in an outpatient setting, Fagan et al. [26] found that though inpatient encounters using telephone and ad hoc interpreters, the visit length was longer compared to encounters using in-person interpreters (telephone encounters = 99.9 min and ad hoc encounters = 92.8 min vs. in-person interpreter encounters = 91 min). Similarly, the provider time was longer in telephone and ad hoc interpreter encounters compared to in-person interpreter encounters (telephone encounters = 36.3 min and ad hoc encounters = 34.4 min vs. in-person interpreter encounters = 26.8 min) [26].

In another study examining throughput time, an indicator for ED crowding, Grover et al. [27] found that throughout times were significantly shorter when patients used in-person interpreters (116 min, *p* < 0.0001) compared to telephone interpretation (141 min) and having a bilingual provider for interpretation (153 min). In a surgical procedural setting, one study revealed that while encounters with an in-person interpreter present were shorter, this varied based on the availability of interpreters, and at times, remote interpretation modalities were conveniently accessed to ensure all language needs were met [28].

#### 3.3.2. Visit Length and Throughput Times

Risk communication before undergoing a surgical procedure is crucial to allow patients to understand the reasons for undergoing surgery, the associated risks of the surgical procedure and to communicate any concerns to clinicians. Only one study from the US examined the use of interpreter service on informed consent for LEP patients [29]. In a pre–post bedside interpreter phone intervention, Lee et al. [29] found that for 68 LEP patients enrolled in the post-implementation group, they were significantly more likely to receive adequate informed consent compared to 84 LEP patients in the pre-implementation group (54% vs. 29%, *p* = 0.001). Furthermore, after adjusting the propensity score, the odds of receiving adequate informed consent were higher for the post-implementation group in the three major informed consent elements: understanding the reasons for surgical procedure (AOR: 3.60; 95% CI (1.52–8.56)), the risks associated with the procedure (AOR: 2.39; 95% CI (1.08–5.29)) and having all questions answered (AOR: 14.1; 95% CI (1.43–139.0)) [29]. However, when compared to 86 English-speaking patients, LEP patients in the post-implementation group were less likely to provide adequate informed consent [29].

#### 3.3.3. Discharge Process

The hospital discharge process is a critical time-point where patients receive essential discharge education and instructions related to care management and medication dosing. Two US studies provided mixed findings concerning the effectiveness of the interpreter service on improving discharge communication for LEP patients [30,31]. While Gutman et al. [30] found that LEP patients who had professional interpretation were likely to receive the complete discharge education from their provider, important discharge contents including medication-dosing education, return precautions and follow-up were missed [30].

In a mixed method study, Lee et al. [31] conducted a pre–post bedside telephone interpreter intervention and used a 15-item care transitions measure to assess patient discharge preparedness. From the 94 patients in the pre-implementation group, and 95 in the post-implementation group, there was no significant difference in overall patient-reported measures of discharge preparedness (77.2 vs. 78.5; *p* = 0.62) [31]. Further findings revealed that patients in the pre-implementation group scored high for medication purpose (88%), and the only significant finding was knowledge of the discharge medication purpose, which increased between the pre- and post- groups (88% vs. 97%, *p* = 0.02) [31]. In the focus group conducted in the second part of the study, the researchers revealed that the non-significant findings may be attributed to clinician preference of using ad hoc interpreters [31].

#### 3.3.4. Treatment and Clinical Care Management

Six studies demonstrated that using professional interpretation for LEP patients with a language barrier increased their access to quality treatment and care for chronic health conditions [32,33,34,35,36,37]. In one US study that examined interpreter use and the quality of acute pain treatment, the researchers found that patients who received interpreter services were significantly likely to have higher levels of pain control and timely pain treatment (*p* = 0.02), and perceived provider helpfulness for pain treatment (*p* = 0.005) [34].

One US study that focused on diabetes care found that the use of professional interpreters increased the likelihood of LEP patients receiving quality diabetes care that met the American Diabetes Association Guidelines, including having two or more clinic visits per year (OR: 2.6; 95% CI: 1.2–5.4), and having one or more dietary consultations (OR: 2.8; 95% CI: 1.3–6.1) compared to English-speaking patients (*p* < 0.05) [37]. Similar findings were found in an Australian study, where 47 LEP patients who identified as requiring interpreter services in a psychiatric inpatient unit were more likely to undergo more consultant reviews (*p* = 0.036); however, this was without a discharge diagnosis [32].

Focusing on stroke rehabilitation care, three studies demonstrated that access to interpreter services improved the quality of stroke care for LEP patients. One US study [36] and two Australian studies [32,34] found that patients with professional interpreters were more likely to receive high quality stroke care compared to those without interpretation. Patients without professional interpretation were less likely to receive documentation related to the contents of stroke education and rehabilitation [35].

#### 3.3.5. Hospital Resource Utilisation

In the ED setting, three US studies illustrated that interpreter service usage had an impact on the likelihood of utilising hospital resources [38,39,40]. Bernstein et al. [38] found that LEP patients receiving interpretation had more primary care and specialty clinic referrals, were more likely to adhere to follow-up visits and were less likely to be readmitted to the ED. In contrast, LEP patients receiving no interpretation had the lowest cost charges of ED visits and return visits compared to LEP patients with interpreted encounters and English-speaking patients (USD $5303 vs. USD $7584 vs. USD $8724, respectively) [38]. Another study found that LEP patients without interpreter use were more likely to receive expensive diagnostic testing (OR +USD 5.78; 95% CI: USD 0.24–USD 11.21) and more frequent hospital admissions (OR: 2.6; 95% CI: 1.4–4.5) [38].

Hartford et al. [40] found that regardless of interpreter service usage, patients with a LEP status were likely to be transferred to the ICU within 24 h of admission compared to English-speaking patients [40]. The researchers suggested that language barriers and interpretation quality might be the reasons for the findings which impact ED assessments, and signs of clinical severity might be missed [40].

#### 3.3.6. Hospital Length of Stay and Readmission Rates

Length of stay (LOS) and readmission rates are quality indicators that assess the overall hospital care performance. Five included studies collected hospital administrative patient data to observe the patterns of LOS and readmission rates of patients provided with professional interpreter services [41,42,43,44,45].

##### Length of Stay

Studies that focused on the impact of the provision of interpreter services on LOS reported complex findings. One longitudinal study from Australia found a significant negative correlation between LOS and the staffing of interpreter services which suggested that as staffing increased for interpreter services, patient LOS decreased [41]. Two studies illustrated that the provision of professional interpreter services at different time-points of hospital admission and discharge had an impact on LOS [42,43].

One Australian study by Abbato et al. [42] found that LOS was significantly shorter when professional interpreter services were provided only in the ED but not provided at either the ED or the inpatient ward (incidence ratio rates (IRR: 0.41; 95%CI: (0.31–0.55); *p* < 0.0001) [42]. In contrast, LOS was significantly longer when professional interpreter services were only provided in the inpatient ward, but not in the ED (IRR: 2.22; 95% CI: (1.76–2.82); *p* < 0.0001)) [42]. Another study from the US showcased similar patterns in which LOS was significantly longer when professional interpreter services were only provided at discharge but not at admission (*p* < 0.01) [43].

In the inpatient setting, Lopez et al. [44] discovered that LOS was the longest for LEP patients who had a physician using professional interpreter services (7.3 ± 7.5). In particular, the Charlson comorbidity score was the highest for LEP patients who had a physician utilising professional interpreter services (2.8 ± 2.6) [44]. This would suggest that physicians would be selective in their care for patients with severe conditions [44].

##### Readmission Rates

Regarding the impact on readmission rates, two US studies found that the provision of interpreter service reduced readmission rates. In a retrospective study, Lindholm et al. [43] found that patients without interpreter use at both admission and discharge had higher readmission rates (24.3%) within 30 days compared to interpreter service usage at both admission and discharge (14.9%) [43]. In a pre–post-intervention study, Karliner et al. [45] also found a decrease in readmission rates during the intervention period, but this was not maintained in the post-intervention period. In contrast, three studies did not have findings associated with the provision of interpreter services and readmission rates in which outcome factors were not fully captured on the hospital administrative system [41,42,44].

#### 3.3.7. Patient Satisfaction

Different types of interpretation modalities have been demonstrated in studies to impact on patient satisfaction in clinical encounters.

##### Face-to-Face Interpretation (In-Person and Videoconferencing)

Face-to-face interpretation is described to be the most preferred type of interpretation which is either delivered by a professional in-person interpreter or through videoconferencing [23,46,47,48]. While studies have reported that professional in-person interpreters received the highest ratings on patient satisfaction [46,47], with advancing technology, videoconferencing has been demonstrated to improve patient satisfaction, yielding similar effects to in-person interpreters [23,48].

In one Australian study, Schulz et al. [48] compared videoconferencing to in-person interpreters and reported that 16% of patients found videoconferencing better or much better, 58% considered both modalities the same and 24% considered videoconferencing worse or much worse. In contrast, when compared to telephone interpretation, 82% of patients considered videoconferencing as better or much better, 15% thought that the modalities were the same and 3% considered it to be worse [48]. One study from the US also provided evidence in patients’ preference in using face-to-face interpretation in family-centered rounds [23]. Anttila et al. [23] found that families using videoconferencing via iPad were significantly more satisfied with their interpretation compared to families using telephone interpretation during and after family-centered rounds (*p* < 0.05). Technical problems have been identified in studies and could create a barrier to increasing access to remote interpretation; therefore, appropriate resources should be available for successful implementation [47,48].

##### Telephone Interpretation

To increase wider access to professional interpretation, two US studies implemented the telephone interpreter service and demonstrated improvements in patient satisfaction [49,50]. Gany et al. [49] compared RSMI to the usual interpreter service in the hospital delivered by either ad hoc interpreters or in-person interpreters. The researchers found that LEP patients who used RSMI were significantly more satisfied with the service where they felt their privacy was being protected and respected by physicians compared to the usual interpreter services (70% vs. 57%; *p* < 0.05) [49].

In a paediatric hospital setting, Cunningham et al. [50] conducted a cohort study that compared telephone interpretation to ad hoc interpreters. The researchers found that LEP mothers who received telephone interpretation were significantly more satisfied with the visit compared to mothers who had ad hoc interpretation (85% vs. 57%, *p* < 0.05) [50]. Furthermore, when compared to ad hoc interpretation, LEP mothers who used telephonic interpreters reported that communication with the physician was “very easy” and they understood all the information when the physician explained it to them (80% vs. 97%, *p* < 0.05) [50].

##### Professional Interpreters vs. Bilingual Providers

To ensure language concordant care for patients with a language barrier, bilingual providers are used to assist with interpretation even when professional interpreters are available. Two US studies found that interpreter service usage had no effect on patient satisfaction when compared to bilingual providers [24,51]. In a paediatric ED setting, Crossman et al. [24] randomised parents into three groups: in-person interpreter cohort, telephone interpretation cohort and bilingual provider cohort. The researchers reported high levels of satisfaction from all three groups; however, a closer examination revealed that the in-person interpreter cohort had worse scores compared to the two cohorts (*p* < 0.001) [24]. While the reason for this finding remained unknown, the researchers suggested that the study might be “overpowered”, or the in-person interpreter was less respectful during the interview process [24].

In a prospective intervention study, Jacobs et al. [51] compared an enhanced interpreter service intervention to the usual hospital interpreter service on patient satisfaction of Spanish-speaking patients. The enhanced interpreter intervention consisted of trained medical interpreters who completed a 120 h internship, whereas the usual hospital interpreter service was delivered by ad hoc interpreters or bilingual providers or hospital interpreters with limited training [51]. Overall, there were no significant findings amongst interpretation groups to suggest that the enhanced interpreter intervention had an impact on patient satisfaction [51].

##### Professional Interpretation vs. No Interpreter Use vs. Ad Hoc Interpreters

Despite the different preferences of interpretation modalities, three studies demonstrated that professional interpretation had a positive impact on patient satisfaction, and the importance of using professional interpreter services instead of using ad hoc interpreters or no interpreter use [52,53,54]. As part of a state-wide evaluation program, one US study examined the provision of interpreter services and patient satisfaction with overall ambulatory care [52]. In this cross-sectional cohort study, Moreno et al. [52] found that patients always using interpreter services were associated with an increase in satisfaction and overall care experience compared to patients who needed interpretation but did not receive it. Another US study demonstrated high levels of patient satisfaction when patients received professional interpretation (92.4%) compared to those receiving ad hoc interpreters, including family members or friends (85.1%) or untrained hospital employees (40%) [53].

The use of professional interpreters has been described as cultural mediators for immigrants or ethnic minorities who do not share the same language as the host country [54]. One Swedish study by Bischoff et al. [54] found that the ratings on patient satisfaction were the highest when professional interpreters were present in clinical encounters. In particular, the researchers further discovered that in patient–provider gender discordant encounters, levels of satisfaction were the lowest when professional interpreters were not used [54].

### 3.4. Theme Three: Cost

This theme presents the cost associated with the provision of interpreter services as described in Table 5.

#### Cost of Interpreter Services

Limited studies have conducted formal cost–benefit or cost-effective analyses associated with the provision of interpreter services in hospitals. One US study identified in this review investigated the provision of a shared network of interpreter services at a low cost [55]. The researchers accounted for a range of data sources to be included for cost calculations which included hospital expenditures and duration of interpreted encounters.

The findings revealed that the most expensive encounters involved rarely encountered languages [55]. When comparing the cost between interpretation modalities, this varied based on the contractual agreement with interpreters and the interpreter service provider. For instance, when considering the cost for in-person interpreters, the cost varied based on whether the interpreter was contracted or an employee at the hospital and would usually require a minimum payment even for only a short encounter [55].

Similarly, the cost for remote interpretation modalities (telephone interpreting and videoconferencing) would also involve a minimum payment depending on the interpreter service provider [55]. For instance, when the cost for videoconferencing was USD $1.00–$3.45 per minute, and a duration of 10.6 min, the cost would be USD 10.60–36.57. With a minimum charge of fifteen minutes, the minimum cost for videoconferencing would be USD 15.00–51.75. The cost information presented in this study provided a guide for hospital institutions and policymakers to determine a cost-saving approach to providing interpreter services to serve the diverse linguistic needs of population groups [55].

## 4. Discussion

With the growing cultural and linguistic diversity among migrants in developed countries, overcoming language barriers in the hospital setting should be a priority to eliminate health disparities. A strategic approach is the provision of professional interpreter services; however, ad hoc interpreters are frequently used by healthcare providers. The findings of this integrative review highlight the importance of using professional interpreter services in the hospital setting to improve communication quality and hospital care for patients with a language barrier.

### 4.1. Communication Quality

Clear and effective communication between patients and healthcare providers is crucial in clinical encounters to ensure all information is accurately conveyed and comprehensible to patients. Concerns regarding the quality and use of interpreter services have been voiced by patients and healthcare providers which have resulted in their reluctance to use the services. The findings of this review suggested that using professional interpretation reduced interpretation errors that have potential clinical consequences [14,15]. Furthermore, comprehension studies suggested that the use of professional interpretation could improve understanding of discharge diagnoses, which is particularly important for parents or caregivers with a language barrier [23,24,25].

While the evidence showcased that different interpretation modalities varied in interpretation accuracy, a consensus finding indicated that using ad hoc interpreters or going without interpreter use altogether when the patient needed one increased interpretation errors [14,15,22]. A study found that professional interpreters who trained more than 100 h had fewer inaccurate utterances compared to their years of experience, and this might have potential policy implications to enhance the professional development of in-person interpreters [14].

### 4.2. Hospital Care Outcomes

Safe and quality care is crucial in the hospital setting to deliver timely and effective care to patients; therefore, the use of interpreter services could shorten the visit length and throughput times to alleviate hospital crowdedness [26,27]. However, one type of interpretation is not sufficient to meet the language needs of linguistically diverse patients, particularly when in-person interpreters are not readily accessible [28]. Remote interpretation modalities should be available such as videoconferencing, which yields similar effects to in-person interpreters with improved patient satisfaction [23,48]. Understanding the purpose of different types of interpretation is crucial to allow wider access to professional interpretation in hospital services.

The planning of interpreter services requires collaborative efforts from policymakers and hospital administrators. Areas that require further examination regarding the hospital care process include risk communication for informed consent and discharge communication. The findings of these studies highlighted the complexity of using professional interpreter services to communicate important informed consent elements and discharge contents relating to medication doses, return precautions and treatment follow-up [29,30,31]. These processes of care require clear and concise communication between the patient and healthcare providers, and to address these issues in the hospital care process, hospital guidelines and instructions to working with interpreters should be available and accessible to healthcare providers.

Complex findings related to LOS were evident in this review. Interpreter service usage at different time-points at hospital admission and discharge have been demonstrated to impact LOS. The findings from two studies suggested that when interpreter services were engaged in the ED or at admission, LOS was shorter compared to only using interpreter services in the inpatient ward or at discharge [42,43]. This may benefit patients and hospitals with lower risks to patient safety and potential cost savings through a shorter LOS [42,43]. From another perspective, a longer LOS may be associated with more timely care delivered to patients [44].

Regarding the impact of interpreter service usage and readmission rates, only one retrospective study found that patients without interpreter service usage at both admission and discharge were more likely to be readmitted [43]. Another pre–post-intervention study also observed a decrease in readmission rates during the intervention period [45]. These studies would suggest that interpreter service usage reduced readmission rates; however, data on interpreter service usage need to be routinely captured on the hospital system for best practice and service evaluation purposes [41,42,44].

### 4.3. Cost of Interpreter Service Usage

The cost of interpreter service provision remains a key consideration for wider implementation in hospitals. The research has shown limited formal cost-effectiveness analyses regarding the use of professional interpreter services in hospitals where cost information is restricted to specific service providers and institutions [55]. One study provided an overview of cost information regarding interpreter services, which would be useful to guide institutions and policymakers to examine the overall cost of service provision and estimate funding support for future purposes [55]. The evidence demonstrated that language barriers could impact long-term healthcare costs with increasing utilisation of hospital resources and more emergency visits [3]. Therefore, decisions to implement professional interpreter services should consider the long-term costs and benefits for future funding support.

### 4.4. Limitations

Several limitations should be noted. Most studies included in the review were conducted in Australia and the US, and only one from Sweden, which may not be generalisable to other countries and settings. This may be due to studies not being available in the English language or not retrievable. However, the lack of research beyond a small set of countries suggests that further work is needed to assess the generalisability of the findings. Additionally, this lack of research may be due to this review only including studies published in English, therefore possibly narrowing the generalisablity of the findings. Furthermore, while a wide range of databases was searched, the review might have missed studies that were relevant to the topic. There were studies that grouped ad hoc interpretation or bilingual provider with professional interpretation, which masked the effects of professional interpretation. Another limitation was the lack of findings on cost impact from the published literature, which prevented the authors from concluding the cost-effectiveness of professional interpreter services. This information would be useful to inform policymakers and hospital administrators for future funding of interpreter services.

### 4.5. Implications for Future Research

This review provides several recommendations for future practices. Targeted policies are needed to strengthen the use of interpreter services in different clinical situations to ensure optimal hospital care delivery. Hospital institutions can consult with relevant stakeholders, including patients and healthcare providers, to provide a better understanding of the patterns of using different interpretation modalities in clinical encounters. The outcome of the consultation process can help determine the purpose of different interpretation methods, and in turn, inform service allocation to match patient and service needs.

In response to the global COVID-19 pandemic, hospitals are reshaping service delivery by using telecommunications, and language services should also be included in this service reform [56]. Remote interpretation modalities (telephone and videoconferencing) are increasingly accessible and reduce direct interactions between patients, healthcare providers and professional interpreters. In particular, videoconferencing is considered a solution in this pandemic which provides face-to-face interaction and has been largely favoured by both patients and healthcare providers [56]. While the negative aspects of remote interpretation modalities are recognised, adequate investments in resources need to be available to address these issues [11]. As the global population continues to increase, cultural competency should be embedded in healthcare to meet the needs of the linguistically diverse population.

## 5. Conclusions

Bridging the language gap should be a priority to prevent the occurrence of adverse events that impact patient safety and quality of care in the hospital setting. The findings from this integrative review suggest that the provision of professional interpreter services can improve communication quality between patients and healthcare providers and hospital care. Nonetheless, improvements should be made around language services in the hospital setting to enhance service quality by providing accurate interpretation. Research on this topic requires efforts from the hospital administrative system to document full records of service utilisation to gain insight into the changing patterns on the outcomes of hospital care.

## Figures and Tables

**Table 1:** Inclusion and exclusion criteria to assess articles.

Inclusion and Exclusion Criteria	
Participants/patients/place	Inclusion: Patients or family members/caregivers with a language barrier presented in their clinical visits and must be in a hospital setting. Exclusion: Patients with a hearing disability or any patient visits that are not in a hospital setting (e.g., community health services).
Interventions	Inclusion: Types of interpretation interventions (i.e., professional in-person interpreters: medical, clinically trained, telephone and videoconferencing interpreter services). No restriction on the duration and frequency of the use of hospital interpreter services. Exclusion: Untrained bilingual providers or hospital interpreter services that are not delivered by a professional interpreter, sign. Translation or written interpreter services.
Comparison group	Different types of interpretation modalities as mentioned above, bilingual providers, ad hoc interpreters and no interpreter use.
Outcomes	Any hospital care and patient outcomes related to the quality of care, patient safety, hospital length of stay, readmissions, satisfaction and hospital cost associated with interpreter service provision.
Study design	Inclusion: Quantitative and mixed-methods study designs. Exclusion: Case studies, reports and reviews, dissertations or qualitative study designs.

**Table 2:** Hospital care outcomes and subcategories.

Themes	Subcategories
Communication quality between patients and healthcare providers	Interpretation errors Patient comprehension
Hospital care outcomes	Throughput times and visit length Informed consent Discharge preparedness Treatment and care management Hospital resource utilization Length of hospital stay and readmissions Patient satisfaction
Cost	Cost of interpreter service provision

**Table 3:** Interpreter service usage and the communication quality between patients and healthcare providers.

Author, Year and Country	Hospital Setting ^1^ and Study Design ^2^	Type of Interpreter Service/ Comparison Group	Sample Characteristics	Outcome(s)	Key Findings
**Interpretation errors**					
Flores, 2012 [14] US	Paediatric ED in Massachusetts ^1^ Cross-sectional study ^2^	Professional interpreters vs. ad hoc interpreters vs. no interpreter use	57 encounters with patients/caregivers with LEP–20 used professional interpreters; 27 used ad hoc interpreters; 10 with no interpreter use **Primary language spoken** Spanish	Medical interpretation errors and clinical consequences: audiotaped encounters and transcript analysis	**Interpretation errors** “Omission” and “false fluency” errors were significantly more likely to be committed by ad hoc interpreters and no interpreter use Omission (*p* = 0.001): ad hoc (46.3%); no interpreter use (54.2%); professional interpreter (41.9%) False fluency (*p* < 0.01): ad hoc (31.6%); no interpreter use (35.9%); professional interpreter (13.6%) **Errors with clinical significance** Lowest for professional interpreters (12%); no interpreter use (20%); highest for ad hoc interpreters (22%) Professional interpreters with >100 training hours had a lower proportion of errors committed compared to interpreters with < 100 training hours (2% vs. 12%, *p* = 0.03)
Nápoles, 2015 [15] US	A public hospital internal medicine clinic ^1^ Cross-sectional study ^2^	Professional interpreter service (in-person professional interpreter and videoconferencing) vs. ad hoc interpreters **Primary language spoken** Spanish	32 encounters from LEP patients; 5 used professional in-person interpreters; 22 used videoconferencing; 5 used ad hoc interpreters **Primary language spoken** Spanish	Interpretation errors and potential clinical significance	**Interpretation errors** Professional interpretation had the least interpretation errors and potential clinical consequence compared to ad hoc interpretation Ad hoc interpretation committed the highest interpretation errors (54%), followed by in-person interpreters (25%) and videoconferencing (23%) Omission was the most common type of error committed (*p* < 0.001); 33% from ad hoc interpreters, and 16% from both in-person interpreters, and videoconferencing **Errors with clinical significance** Clinically significant errors occurred mostly in visits with ad hoc interpreters (8%), visits using videoconferencing (7%) and visits with in-person interpreters (3%)
Flores, 2003 [20] US	Hospital outpatient clinic ^1^ Quantitative—Not specified ^2^	Professional hospital interpreters vs. ad hoc interpreters	Audiotaped 13 clinical encounters with an interpreter present **Primary language spoken** Spanish	Interpretation errors and clinical significance	**Interpretation errors** False fluency occurred more in encounters with hospital interpreters compared to encounters with ad hoc interpreters (22% vs. 9%, *p* = 0.001) 76% of false fluency errors were committed by healthcare providers (58% occurred when the interpreter was not in the room or interpretation on the phone; 42% of errors were made by the provider without any correction by the interpreter) **Clinical significance** Errors with clinical significance were significantly likely to occur in encounters with ad hoc interpreters compared to hospital interpreters (77% vs. 53%, *p* < 0.001)
Gany, 2007 [21] US	Audiotaped transcripts of primary cases ^1^ Quantitative—Not specified ^2^	Remote simultaneous medical interpretation (RSMI) vs. remote consecutive, proximate consecutive (in-person interpreter), ad hoc interpreter	16 encounters yielded 1909 utterances	Interpretation errors	**Interpretation errors** Non-RSMI interpreting resulted in 12 times more medical errors compared to RSMI (*p* = 0.0002)
Hornberger, 1996 [22] US	Wellbaby clinic of a hospital ^1^ RCT ^2^	RSMI vs. proximate consecutive interpretation (in-person interpretation)	27 LEP mothers attended scheduled well-baby visits; 13 received in-person interpretation; 14 received RSMI intervention	Quality of interpretation and preference of interpretation	**Interpretation Quality** RSMI had lower rate (13%) of inaccurate interpreted utterances compared to in-person interpretation; omission errors were the frequently committed errors **Interpretation preference** Mothers preferred the experimental intervention compared to the in-person interpretation
**Comprehension**					
Anttila, 2017 [23] US	Tertiary care paediatric hospital ^1^ Observational study ^2^	Professional interpreter services (certified in-person medical interpreter, certified bilingual physician, telephone interpretation and videoconferencing via iPad) vs. ad hoc interpretation (family member)	124 Spanish-speaking families: 29 used a certified medical interpreter; 22 used a certified bilingual provider; 26 used telephone interpretation; 7 for videoconferencing **Primary language spoken** Spanish	Efficacy of interpretation type	**Efficacy of interpretation type** Significant difference in caregivers’ comprehension between the modes of interpretation (*p* = 0.01) All caregivers that used videoconferencing reported a “complete” understanding of child’s condition; 90% of in-person interpreter users felt the same; 58% and 50% of families reported a “complete” understanding of their child’s condition when using telephone interpretation during and after the visit
Crossman, 2010 [24] US	Urban paediatric ED ^1^ Prospective, randomised trial ^2^	Telephone and in-person interpretation vs. bilingual providers	1201 families were enrolled: 407 used telephone interpretation; 377 used in-person interpreters; 417 had a bilingual provider	Family comprehension and satisfaction	**Family comprehension** No difference in family comprehension of child’s admission or discharge diagnosis amongst interpretation groups (telephone 95.1%; in-person 95.5%; bilingual 95.4%)
Lion, 2015 [25] US	Paediatric ED ^1^ RCT ^2^	Remote interpretation modalities (telephone interpretation vs. videoconferencing)	LEP patients—107 in the telephone group and 142 in the video group	Comprehension of child’s diagnosis; communication and interpretation quality	**Comprehension** Parents in the video group were significantly more likely to name their child’s diagnosis compared to the telephone group (74.6% vs. 59.8%; *p* = 0.03) **Communication quality** No difference in communication and interpretation quality between the two remote modalities

^1^ Hospital setting. ^2^ Study design.

**Table 4:** Summary of studies that examine the impact on hospital care outcomes.

Author, Year and Country	Hospital Setting ^1^ and Study Design ^2^	Type of Interpreter Service/ Comparison Group	Sample Characteristics	Outcome(s)	Key Findings
**Visit length and throughput times**					
Fagan, 2003 [26] US	Hospital-based outpatient clinic ^1^ Time motion study ^2^	Hospital interpreter (trained and certified) and telephone interpretation	LEP patients; 51 used hospital interpreters; 31 used telephone; 90 used patient-supplied interpreter **Primary language spoken** Spanish	Visit length and provider time	**Visit length** Significantly longer when LEP patients used some form of interpretation compared to patients without interpretation (93.6 min vs. 82.4 min, *p* = 0.002) and provider times (32.4 min vs. 28 min, *p* < 0.001) **Telephone interpretation vs. no interpreter use:** significantly longer mean clinic times (99.9 min vs. 82.4 min, *p* = 0.02) and provider times (36.3 min vs. 28 min, *p* < 0.001) **Ad hoc interpreter vs. no interpreter use:** significantly longer mean clinic times (92.8 min vs. 82.4 min, *p* = 0.027) and provider times (34.4 min vs. 28 min, *p* < 0.001) **Professional interpreter vs. no interpreter use:** no significant difference in mean clinic times (91 min vs. 82.4 min, *p* = 0.16) and mean provider times (26.8 min vs. 28.0 min, *p* = 0.51)
Grover, 2012 [27] US	Paediatric ED ^1^ Prospective, secondary analysis ^2^	In-person professional vs. telephonic interpretation vs. bilingual provider	1196 families with LEP: 404 used telephonic interpretation; 375 used in-person interpreter; 417 bilingual **Primary language spoken** Spanish	Throughput time	**Throughput time** Shorter throughput time for professional in-person interpreters compared to telephonic interpretation and bilingual providers (116 min vs. 141 min vs. 153 min, *p* < 0.0001)
Burkle, 2017 [28] US	Surgical and procedural floors ^1^ Quantitative—Not specified ^2^	Language services (in-person, telephone and video interpretation)	A total of 318 LEP patient records: 241 in-person, 55 telephone and 9 in video interpretation	Efficiency of interpreter services	**Efficiency** The mean arrival time for in-person professional interpreter service was 19 min; however, this varied based on the availability of in-person interpreters. The use of remote modalities resulted in no delay and cancellation of interpretation services
**Informed consent**					
Lee, 2017 [29] US	Cardiovascular, general surgery or orthopaedic surgery floors ^1^ Prospective, pre-post ^2^	Bedside interpreter phone	152 LEP patients: 84 pre- 68 post-implementation	Patient evaluation of informed consent (survey)	**Informed consent** LEP patients were significantly likely to receive adequate informed consent compared in the pre-implementation stage (54% vs. 29%, *p* = 0.001); higher odds of understanding the reasons for their procedure (adjusted odd ratio—3.60; 95% CI = 1.08–5.29), the risks associated with the procedure (AOR = 2.39; 95% CI = 1.08–5.29) and had all their questions answered (AOR = 14.1; 95% CI = 1.43–139)
**Discharge outcomes**					
Gutman, 2018 [30] US	Paediatric ED ^1^ RCT ^2^	Professional interpretation services (telephone and video) vs. bilingual provider	47 caregivers with LEP 66% used professional interpreters and 3% had a bilingual provider as interpreter **Primary language spoken** Spanish	Discharge preparedness	**Discharge preparedness** LEP patients that used professional interpretation compared to no interpreter use had increased odds of receiving complete discharge education (odds ratio = 7.1; 95% CI = 1.4–37), and increased odds of high-quality assessment for caregiver comprehension by the provider (OR = 6.1; 95% CI = 2.3–15.9) Important discharge contents regarding medication dosing, return precautions and follow-up treatment were missed
Lee, 2018 [31] US	Cardiovascular, general surgery or orthopaedic surgery floors ^1^ Mixed-methods (survey and focus group) ^2^	Bedside interpreter phone	189 LEP patients: 94 pre- and 95 post-implementation	Discharge preparedness	**Discharge preparedness** No significant difference in pre-and post-discharge preparedness (*p* = 0.62) Only significant finding was an increased knowledge of discharge medication purpose between pre- and post-intervention (*p* = 0.02) In a focus group discussion with physicians and nurses, they preferred in-person interpreters to communicate complex discharge contents
**Treatment and clinical care management**					
Daly, 2019 [32] Australia	Inpatient psychiatric unit ^1^ Retrospective study ^2^	Interpreter service usage vs. English-speaking patients	Total of 47 LEP patients who required interpreter service and 47 English-speaking patients	Clinical outcomes	**Clinical outcomes** LEP patients underwent more consultant reviews (*p* = 0.036) but attracted different diagnoses with no discharge diagnosis made (*p* = 0.018)
Davies, 2016 [33] Australia	Inpatient setting of two rehabilitation hospitals ^1^ Retrospective case-control study ^2^	Interpreter service use (low English proficiency group) vs. high English proficiency group	Low English proficiency group (comprised of LEP patients whose preferred language was not English or accessed to interpreter service) **Primary language spoken** Arabic, Turkish, Italian, Greek, Macedonian, Assyrian and Chaldean, Vietnamese and Chinese	Diabetes care—FIM (functional improvement measure)	**Diabetes care** Significant differences in FIM efficiency were found between interpreter service usage and without interpreter use (FIM efficiency, *p* = 0.01; and FIM motor efficiency, *p* = 0.04)
Jimenez, 2012 [34] US	Obstetric and gynaecological unit of a teaching hospital ^1^ Secondary analysis—cross-sectional surveys ^2^	Professional interpreter service (state-wide program) vs. no interpreter use	27% of patients always received an interpreter, and 73% sometimes (not always) received an interpreter **Primary language spoken** Not specified	Quality of pain treatment	**Treatment outcome** Quality of pain control was higher for patients who always received interpreters (*p* = 0.02), timely pain treatment (*p* = 0.02) and perceived provider helpfulness to treat their pain (*p* = 0.005) compared to patients without frequent interpreter usage
Kilkenny, 2018 [35] Australia	Data collected from the Australian Stroke Clinical Registry (AuSCR) from 45 hospitals ^1^ Retrospective study ^2^	Use of interpreter service vs. no interpreter use	A total of 1461 of 34,562 (4.2%) patients required an interpreter—older patients had greater severity of the stroke, and took longer to arrive at the hospital **Primary language spoken** Not specified	Stroke care	**Stroke care outcomes** Patients requiring an interpreter more often received care on a stroke unit (85% versus 78%; *p* < 0.001) than those not requiring an interpreter, while all other processes of care remained similar
Luan, 2017 [36] US	GTWG-Stroke (Get with the Guidelines–Stroke) Registry at Massachusetts General Hospital ^1^ Retrospective study ^2^	Professional medical interpreters vs. no interpreter use	259 LEP patients: 147 used a professional medical interpreter; 112 did not use an interpreter **Primary language spoken** Spanish, Portuguese, French/Haitian Creole, Mandarin/Cantonese	Quality of acute ischemic stroke (AIS) care	**Stroke care outcomes** LEP patients without interpreter use were less likely to receive detect-free AIS care compared to those receiving professional interpretation (OR: 0.50; 95% CI: 0.27–0.90; *p* = 0.02) More specifically, contents of stroke education and consideration for rehabilitation were not documented for LEP patients without language assistance
Tocher, 1998 [37] US	Primary and specialty care clinics at a university and a county hospital ^1^ Comparative study ^2^	Professional interpreter vs. English-speaking patients	93 LEP patients with type 2 diabetes who all used professional interpreters, and 529 English-speaking patients **Primary language spoken** Spanish, Russian, Cambodian, Vietnamese, Tigrinya	Process and outcome of diabetes care (based on the American Diabetes Association ADA guidelines), including having two or more standardised glycohaemoglobin test or physician visits or dietary consultations	**Outcomes of diabetes care** Overall provision of professional interpreters improved diabetes care that met the ADA guidelines for LEP patients with type 2 diabetes; significantly likely to receive standardised glycohaemoglobin test or more than two physician visits per year (*p* < 0.05); and more likely than English speakers to receive one or more dietary consultations (*p* < 0.01)
**Hospital resource utilisation**					
Bernstein, 2002 [38] US	ED ^1^ Retrospective, descriptive study ^2^	Interpreter service usage vs. no interpreter use vs. English-speaking patients	63 LEP patients with interpreter service usage; 374 LEP patients without interpreter use; 63 English-speaking patients **Primary language spoken** Spanish, Haitian Creole and Portuguese Creole	ED utilisation and utilisation cost	**ED utilisation** LEP patients without interpreter use had the shortest length of visit, and fewer assessment testing and procedures. Professional interpreter use was associated with increasing access to primary care and specialty clinic referrals, being more likely to adhere to follow-up visits and less likely to be readmitted to the ED. **Utilisation cost** Both charges for ED visits and returns were the lowest for LEP patients with no interpreter use (USD $5303), followed by patients with interpreter use (USD $7584) and the highest for English-speaking patients (USD $8724)
Hampers, 2002 [39] US	Paediatric ED ^1^ Cohort study ^2^	Professional interpreter (interpreters underwent a minimum of 40 h training) vs. no interpreter use vs. bilingual provider vs. English-speaking patients	Total of 4146 visits: 550 families with LEP; 239 encounters with a professional interpreter; 141 encounters without interpreter use; 170 encounters used a bilingual provider **Primary language spoken** Spanish, Polish, Russian, Vietnamese	ED resource utilization and associated cost	**ED resource utilisation** Bilingual cohort had similar rates of resource utilisation as English-speaking patients **Professional interpreter cohort:** more likely to be admitted (OR: 1.7; 95% CI [1.1–2.8]; least likely to be tested (OR: 0.73; 95% CI [0.56–0.97]) but with longer ED visit length (+16 min; 95%CI [6.2–26 min] **No-interpretation cohort:** more likely to be tested (OR: 1.5; 95%CI [1.04–2.2] and receive expensive testing cost (+USD 5.78; 95%CI (USD 0.24–11.21); most likely to be admitted (OR = 2.6; 95%CI (1.4–4.5) but no difference in ED visit length
Hartford, 2019 [40] US	Paediatric ED ^1^ Retrospective cohort study ^2^	Videoconferencing vs. in-person interpreters vs. telephone interpretation	LEP patients: 51.6% received videoconferencing; 15.3% received in-person interpreters; and 9.7% telephone interpretation; 23.4% used multiple interpretation modalities **Primary language spoken** Spanish, Somali, Cantonese or Mandarin, Vietnamese, Amharic, Arabic, Oromo, Tigrinya and Russian	ED LOS, ICU admissions and return visits	**ED LOS** Shortest LOS for LEP patients without interpretation (186.18 min) and the longest for those receiving interpretation (210.45) **ICU admissions** LEP patients without interpretation were less likely to be admitted than EP patients (OR 0.69, 95% [0.62−0.78]); when LEP patients received interpretation, their odds of admission were slightly higher than EP patients (OR 1.12, 95% CI) [1.01−1.25]. **Return visits** No difference in return visits when comparing EP to LEP with or without interpretation
**Hospital length of stay and readmissions**					
Beagley, 2020 [41] Australia	Large metropolitan public healthcare facility ^1^ Longitudinal study presenting data over a 10-year period ^2^	Interpreter-mediated encounters vs. encounters without interpretation	Non-English-speaking patients (NESP) vs. English-speaking patients	LOS and readmission rates	**LOS** LOS was significantly negatively correlated with TALS staffing, suggesting that LEP patient (NESP) LOS decreased as interpreter staffing increased **Readmission rates** No significant finding
Abbato, 2019 [42] Australia	Emergency department (ED) and inpatient wards ^1^ Retrospective audit ^2^	Professional interpreter services vs. no interpreter used	448 LEP patients: 93 patients (21%) received professional interpretation in the ED and 116 patients (26%) received professional interpretation in the inpatient ward **Primary language spoken** Greek, Vietnamese, Mandarin, Farsi/Persian and Spanish	Length of stay (LOS) and 30-day readmission rates	**ED LOS** Shorter LOS for patients only using professional interpreters in the ED but not either in the ED or the inpatient ward (IRR: 0.41; 95%CI: 0.31–0.55; *p* < 0.0001) **Inpatient LOS** Longer LOS when LEP patients used professional interpreters only in the inpatient ward but not in the ED (IRR: 2.22; 95% CI: (1.76–2.82); *p* < 0.0001) **ED vs. inpatient ward** Mean LOS for patients receiving interpreters in the ED was 19.3 h compared to a mean LOS of 100.2 h for LEP patients using interpreters only in the inpatient ward **30-day readmission** No significant findings for hospital readmissions
Lindholm, 2012 [43] US	Tertiary care, university hospital ^1^ Retrospective analysis ^2^	Professional interpreter service vs. no interpreter use	3071 LEP patients: 39% of LEP patients received interpretation at admission and discharge; 14% without interpreter use at admission or discharge Spanish and Portuguese speakers more likely to receive interpretation at both admission and discharge, whereas patients with less prevalent languages were less likely to receive interpretation **Primary language spoken** Spanish, Portuguese, Vietnamese, Albanian, Russian and others	LOS and 30-day readmission rates	**LOS** Compared to patients using interpretation at both admission and discharge, increased LOS for LEP patients who did not receive professional interpretation by between 0.75 and 1.47 days (*p* < 0.02) A longer LOS was also found in patients only receiving interpretation at discharge but not admission **Readmission rates** Higher readmission rates for patients without interpretation at both admission and discharge (24.3%); 16.9% when professional interpreter was used at admission only; 17.6% when professional interpreter was used at discharge only; and the lowest readmission rates (14.9%) for LEP patients who had professional interpretation at both admission and discharge (Chi-square = 19.5, df = 3, *p* < 0.001)
López, 2015 [44] US	General medicine service at a large tertiary academic hospital ^1^ Retrospective cohort analysis ^2^	Hospital interpreter service (in-person, telephone and video interpretation) vs. English-speaking patients	564 LEP patients: 65.8% had no interpreter use, and 34.2% used hospital interpreter service Patients were categorised into four groups: (1) interpreter use by a non-physician; (2) interpreter use by a non-hospitalist physician; (3) interpreter use by a hospitalist; (4) no interpreter used **Primary language spoken** Not-specified 1963 LEP patients	LOS and readmission rates	**LOS** Using professional interpretation with a physician present had the longest LOS (7.3 ± 7.5); using professional interpretation with a non-physician present had the shortest LOS (4.7 ± 2.6) Patients with interpreter use and a physician present had the highest Charlson score (2.8 ± 2.6), which would suggest that physicians were selective in their care for patients with severe conditions **Readmission rates** No significant finding
Karliner, 2017 [45] US	A medicine floor of an academic hospital ^1^ Natural experiment (pre- and post-intervention) ^2^	Dual handset interpreter telephone at every beside (intervention during the 8-month period) vs. English-speaking patients	Pre-intervention: 4131 patients; intervention: 1714 patients; post-intervention: 2132 **Primary language spoken** Chinese, Russian, Spanish, others (Amharic, Arabic, Cambodian, etc.)	Readmission	**Readmission rates** Readmission rates significantly decreased comparatively during the 8-month duration to pre-intervention (17.8% to 13.4%; *p* = 0.04)
**Patient satisfaction**					
Anttila, 2017 [23] US	Tertiary care paediatric hospital ^1^ Observational study ^2^	Professional interpreter services (certified in-person medical interpreter, certified bilingual physician, telephone interpretation and videoconferencing via iPad) vs. ad hoc interpretation (family member)	124 Spanish-speaking families; 29 used a certified medical interpreter; 22 used a certified bilingual provider; 26 used telephone interpretation; 7 for videoconferencing **Primary language spoken** Spanish	Family satisfaction	**Family satisfaction** Higher satisfaction with videoconferencing via iPad interpretation compared to telephone interpretation during and after family-centered round (*p* < 0.05)
Crossman, 2010 [24] US	Urban paediatric ED ^1^ Prospective, randomised trial ^2^	Telephone and in-person interpretation vs. bilingual providers	1201 families were enrolled: 407 used telephone interpretation; 377 used in-person interpreters; 417 had a bilingual provider	Satisfaction	**Satisfaction** The quality and satisfaction were worse in the in-person cohort compared to the telephone and bilingual cohort (*p* < 0.001) Patients in the bilingual cohort were less satisfied with their language service than those in the in-person and telephone cohorts (*p* < 0.001) No type of interpretation was the best
Bagchi, 2011 [46] US	EDs of two hospitals ^1^ RCT ^2^	Professional in-person interpreter service (treatment time-block) vs. usual interpreter service in the hospital (ad hoc interpreters, telephone interpretation, untrained bilingual providers—control time block) **Primary language spoken** Spanish	531 LEP patients—47 refused, 37 patients excluded as they already participated in the study 242 in the treatment time block group (227 received a professional in-person interpreter, 1 did not receive an interpreter, 17 received the usual interpreter service in the hospital); 205 in the control time block group (66 patients without interpreter use, 114 patients receiving the usual hospital service, 11 likely to receive a bilingual provider)	Patient satisfaction	**Treatment intervention** 96% of patients in the intervention reported to be “very satisfied” with the visit, and 93% found the visit interaction “very easy” to understand **Control group** Only 24% of patients in the control group reported to be “very satisfied” with the visit, 18% reported the visit interaction as “very easy” to understand
Locatis, 2010 [47] US	Post-partum and paediatric clinics of a teaching hospital ^1^ Quasi-randomised control study ^2^	In-person interpreters vs. remote interpretation modalities	241 patients requiring interpreter services; 80 used in-person interpreters; 80 used telephone interpretation; 81 used videoconferencing **Primary language spoken** Spanish	Satisfaction with encounter quality: patients, provider and interpreters (survey)	**Satisfaction outcome** Patients rated all interpretation modes the same Only eleven responded to the communication method: six positive comments for video interpretation, three negatives for telephone interpretation and two positives for in-person interpretation A majority of providers and interpreters preferred in-person interpretation
Schulz, 2015 [48] Australia	The Travel and Immigrant Health Clinic in the Victorian Infectious Diseases Service at Royal Melbourne Hospital ^1^ Quantitative (surveys), not specified ^2^	Video interpretation vs. in-person and telephone interpretation	Refugees who recently settled in Australia: total of 65 occasions with requested interpreter service bookings; 56 interpreter-attended occasions; of these occasions, 47 LEP patients completed surveys **Primary language spoken** Burmese, Karen and Haka Chin	Patient and doctor satisfaction, and practical limitations	**Patient satisfaction** **overall:** 98% of patients were satisfied with videoconferencing **Compared to telephone interpretation:** 82% of patients thought videoconferencing was better, 15% considered both the same and 3% considered videoconferencing worse **Compared to in-person interpreters:** 16% thought videoconferencing was better or much better, 58% considered the same and 24% considered the modality worse Professional in-person interpreters remained the most preferred type of interpreter service
Gany, 2007 [49] US	Primary care clinic and ED at a municipal hospital in New York ^1^ RCT—stratified randomisation ^2^	RSMI (telephonic interpretation) vs. usual hospital interpreter service	735 LEP patients with language discordant encounters: 371 assigned to RSMI; 364 enrolled to the usual service (onsite trained interpreters, excluding ad hoc interpreters) **Primary language spoken** Spanish, Mandarin or Cantonese	Patient satisfaction (questionnaire)	**Patient satisfaction** LEP patients in the RSMI group reported the highest satisfaction, in which they felt respected by their physician compared to those in the in-person interpreter group (70% vs. 57%, *p* < 0.05), and thought their physician understood them (45% vs. 35%, *p* < 0.05) Overall satisfaction with physician care was higher in the RSMI group compared to the in-person interpreter group (*p* < 0.05) RSMI can improve patient satisfaction and protect privacy among LEP patients
Cunningham, 2008 [50] US	An urban university hospital affiliated practice—padiatric ^1^ Cohort study ^2^	Telephone interpretation vs. ad hoc interpreters	98 Spanish-speaking mothers with LEP: 46 relied on ad hoc interpreters; 52 received telephonic interpretation	Patient satisfaction (survey)	**Patient satisfaction** Compared to ad hoc interpretation, mothers who received telephone interpretation reported higher satisfaction with overall clinic visits (57% vs. 85%, *p* < 0.05) and felt it was “very easy” to communicate with the doctor (22% vs. 83%, *p* < 0.01) Overall use of telephonic interpretation service was helpful and improved well-baby visits of LEP mothers
Jacobs, 2007 [51] US	Public hospital inpatient medicine service ^1^ Prospective intervention study ^2^	Enhanced intervention (professional medical interpreters) vs. usual interpreter service (ad hoc interpreters, bilingual interpreters—limited training)	LEP patients: 124 accessed enhanced interpretation and 99 accessed usual interpreter service **Primary language spoken** Spanish	Patient satisfaction	**Patient satisfaction:** the intervention did not have a significant impact on the outcome
Moreno, 2010 [52] US	Outpatient setting across hospital sites in the US ^1^ Cross-sectional cohort study ^2^	Interpreter service usage—patients who needed and always used interpretation vs. those who needed but did not always use an interpreter vs. no interpreter use	1590 patients: 18% patients needed an interpreter but did not always use one; 39% always had interpreters available; 13% needed an interpreter but never had one; others indicated a need for an interpreter and usually or sometimes had one available **Primary language spoken** Spanish	Patient satisfaction; doctor communication and perceived helpfulness of office staff (survey)	**Patient satisfaction** Frequent interpreter usage was associated with greater satisfaction with overall care (*p* < 0.01) and an increase in doctor/staff communication scores (*p* < 0.001) Overall provision of interpreter service improved patient satisfaction in the outpatient setting
Kuo, 1999 [53] US	A medial primary care unit at a hospital ^1^ Quantitative (survey) ^2^	Professional interpreter (telephone interpretation vs. ad hoc interpreters vs. bilingual providers)	149 Spanish-speaking patients: 65% of patients reported frequent use of ad hoc interpreters; 45% used telephone interpretation; 65% used professional interpreters; 77% used a hospital employee; and 20.5% used bilingual providers **Primary language spoken** Spanish	Patient satisfaction	**Patient satisfaction** Professional interpretation received the highest level of satisfaction by patients (92.4%); however, they were significantly more satisfied when family members or friends were used (*p* < 0.01)
Bischoff, 2008 [54] Sweden	Outpatient clinic ^2^ Cross-sectional study ^2^	Doctor–patient gender concordant care: professional interpreter use vs. no interpreter use	A total of 363 clinical encounters with foreign language-speaking patients **Primary language spoken** Albanian, Serbo-Croatian/Bosnian, Somali, Spanish, English, Arabic and Farsi	Doctor–patient gender concordant care and patient satisfaction	**Patient satisfaction** The use of professional interpretation improved patient satisfaction and communication in doctor–patient gender discordant encounters (*p* = 0.01)

^1^ Hospital setting. ^2^ Study design.

**Table 5:** Data sources for cost calculation.

Consideration for Cost Calculation	
Hospital Expenditures	Interpreter Network Admin
Interpreter salaries	Number of interpreted encounters
Bonuses for dual-role interpreters (include nurses, doctors who also served as interpreters)	Durations of interpreted encounters and their usage each month
Manager salaries and time-spent managing the shared network	Languages interpreted
Cost to outsource telephone interpreter service and videoconferencing	Time spent interpreting each month
Annual network fee	The time for interpreters spent either waiting or interpreting
Investment in equipment	

## Data Availability

Not applicable.
